# Near-infrared photoresponsive drug delivery nanosystems for cancer photo-chemotherapy

**DOI:** 10.1186/s12951-020-00668-5

**Published:** 2020-08-03

**Authors:** Xiaoying Wang, Zeliang Xuan, Xiaofeng Zhu, Haitao Sun, Jingchao Li, Zongyu Xie

**Affiliations:** 1 Xuhui District Center for Disease Control and Prevention, Shanghai, 200237 China; 2 Department of Radiology, The First Affiliated Hospital of Bengbu Medical College, Bengbu, 233004 Anhui China; 3 Shanghai Institute of Medical Imaging, Department of Interventional Radiology, Zhongshan Hospital, Fudan University, Shanghai, 200032 China; 4 College of Chemistry, Chemical Engineering and Biotechnology, Donghua University, Shanghai, 201620 China

**Keywords:** Drug delivery systems, Near-infrared light, Nanomaterials, Chemotherapy, Phototherapy

## Abstract

Drug delivery systems (DDSs) based on nanomaterials have shown a promise for cancer chemotherapy; however, it remains a great challenge to localize on-demand release of anticancer drugs in tumor tissues to improve therapeutic effects and minimize the side effects. In this regard, photoresponsive DDSs that employ light as an external stimulus can offer a precise spatiotemporal control of drug release at desired sites of interest. Most photoresponsive DDSs are only responsive to ultraviolet-visible light that shows phototoxicity and/or shallow tissue penetration depth, and thereby their applications are greatly restricted. To address these issues, near-infrared (NIR) photoresponsive DDSs have been developed. In this review, the development of NIR photoresponsive DDSs in last several years for cancer photo-chemotherapy are summarized. They can achieve on-demand release of drugs into tumors of living animals through photothermal, photodynamic, and photoconversion mechanisms, affording obviously amplified therapeutic effects in synergy with phototherapy. Finally, the existing challenges and further perspectives on the development of NIR photoresponsive DDSs and their clinical translation are discussed.

## Introduction

Chemotherapy is one of the most common treatment strategies in the clinic for cancer, which however often has the issues of low therapeutic efficacy, intrinsic drug resistance and severe side effects [1–3]. Although nanomaterial-based drug delivery systems (DDSs) can mitigate the problems [4–7], ineluctable accumulation of therapeutic drugs in healthy tissues is still prominent. In contrast, stimuli-responsive DDSs with controllable on-demand drug release profiles have been demonstrated to allow highly specific cancer treatment with reduced toxic concerns to normal tissues [8–10]. Within the exciting stimuli-responsive DDSs, typical controlled release of drugs mainly relies on some endogenous reactions in the biological systems, such as, cleavages of reactive oxygen species (ROS)-responsive moieties by oxidative stress [11–13], charge reversal of polyelectrolytes in acidic microenvironment [14–16], proteolysis of peptide substrates by enzymes [17–19], cleavages of disulfide linkers under reductive conditions [20–22], and reduction of hypoxia-responsive moieties in hypoxic conditions [23, 24]. However, these DDSs still display an insignificant tumor selectivity due to undesirable release in circulation and off-target release caused by endogenous processes in healthy tissues [25–27].

External stimuli can provide an improved spatiotemporal control of drug release, and thus have received tremendous attention in the field of DDSs [28–30]. In particular, a promising toolbox is the construction of photoresponsive DDSs based on external light that has the advantages of noninvasive nature, ease of production, simplicity of operation, good controllability over both wavelength and intensity, and high spatiotemporal resolution [31–33]. Although there are various light-sensitive moieties that are responsive to ultraviolet (UV)–visible light, their applications in DDSs are restricted by the high phototoxicity of UV light and shallow tissue penetration depths for both UV and visible lights (< 1 mm) [34–36]. In contrast, near-infrared (NIR) light (650–950 nm) displays negligible phototoxicity and can penetrate more deeply into biological tissues (1–3.5 mm) [37]. In the presence of suitable optical materials as the transducers, NIR light can be converted into heat for photothermal therapy (PTT), ROS for photodynamic therapy (PDT), and UV/visible photons for photoregulation [38–40]. Therefore, it is feasible to integrate thermal-, ROS- and short-wavelength light-sensitive components into DDSs to achieve photoresponsive drug release for precise cancer therapy.

Herein, we summarize the recent development of NIR photoresponsive DDSs with on-demand drug release profiles for cancer photo-chemotherapy. They are first classified into (i) photothermal responsive DDSs, (ii) photodynamic responsive DDSs, and (iii) photoconversion responsive DDSs based on three different photoresponsive mechanisms. The constructions, NIR light triggered drug release profiles of these DDSs, and their applications for cancer therapy are then introduced. At last, a brief conclusion and discussion of the existing challenges and further perspectives are given.

## Classification of photoresponsive DDSs

NIR photoresponsive DDSs can be classified into three categories: (i) photothermal responsive DDSs, (ii) photodynamic responsive DDSs, and (iii) photoconversion responsive DDSs according to different mechanisms. For cancer therapy, DDSs are typically administered via intravenous injection, and accumulate into tumor tissues though the enhanced permeability and retention (EPR) effect [41]. The representative NIR photoresponsive DDSs used for synergetic treatments of tumors in living animals are listed in Table 1.

**Table 1:** Summary of representative NIR photoresponsive DDSs for cancer therapy

Classification	DDS	Photosensitive material	NIR light wavelength	Tumor model	Refs
Photothermal responsive DDSs	CuS-DOX-MBA@PCM	Stearic acid, lauric acid	808	EAC	[57]
	DOX/MCN	DPPC	808	4T1	[58]
	PAM/Pt@IcLipo	DPPC	808	4T1	[59]
	DOX/ICG/PCM	Auric acid, stearic acid	808	HeLa	[60]
	IR-780/LON liposome	DPPC	808	LL/2	[61]
	P(DPP-BT/DOX)	Lauric acid, stearic acid	730	HeLa	[62]
	TENAB nanoparticles	Linoleic acid, stearyl alcohol	808	HeLa	[63]
	DOX/DiR/ABC	Ammonium bicarbonate	760	4T1	[65]
	DTX/Au/ABC	Ammonium bicarbonate	808	S180	[66]
	PD@BS	1-Tetradecanol	808	MDA-MB-231	[71]
	Prussian blue/DOX/PCM	1-Pentadecanol	808	HepG2	[72]
	UCNPs@ZrO_2_-Ce6/DOX/PCM	1-Tetradecanol	808	U14	[74]
	HPDC	1-Tetradecano	808	4T1	[75]
Photodynamic responsive DDSs	Biomimetic dimeric prodrug	Thioketal linker	638	HeLa	[86]
	CMSNR-B-PEG/G3-Pt	Bis-(alkylthio)alkene linker	660	4T1	[92]
	PTS-DP	Thioketal linker	670	CT26	[96]
	β-CD/Ada-BODIPY/Ada-PTX	Aminoacrylate group	660	HeLa	[97]
	DOX/CP-NI	2-Nitroimidazole	635	HeLa	[98]
	Ce6/TPZ liposomes	2-Nitroimidazole	670	MCF-7	[99]
	SPN-prodrugs	Hypoxia-cleavable linker	808	4T1	[100]
Photoconversion responsive DDSs	UCNP/ACCh	Coumarin	980	S180	[107]
	TTA-UC nanoparticles	Coumarin	650	4T1	[108]
	UCNP/Pt(IV)	Pt(IV)	980	H22	[110]
	UCNP-Pt(IV)-polymer	Pt(IV)	980	U14	[112]
	UCNPs/DOX-TAT-HA	Azobenzene	980	HepG2	[114]
	UCNP-curcumin	Spiropyran	980	4T1	[115]
	UCNP-RB-polymer	2-Nitrobenzyl	980	TT	[116]

**Fig. 1:**
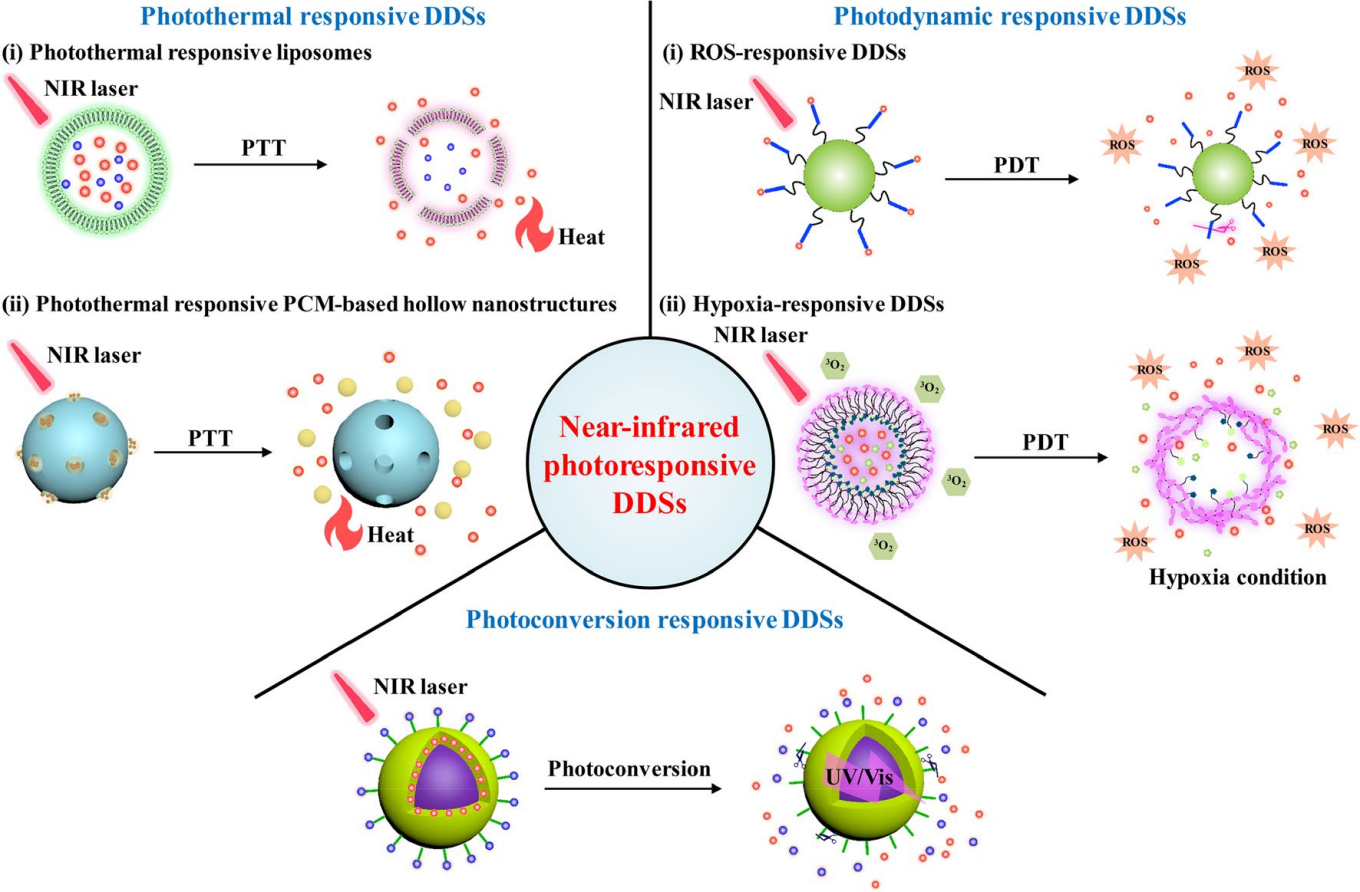
Schematic illustration of NIR photoresponsive DDSs

Photothermal responsive DDSs achieve on-demand release of drugs via nanomaterial-mediated PTT under NIR laser irradiation to generate heat to destroy thermal-responsive materials (Fig. 1) [42]. Such a class of DDSs are generally constructed via integrating thermal-responsive components into nanomaterials containing drugs and photothermal agents [43, 44]. Due to the excellent photothermal conversion efficacy, NIR-absorbing dyes, polydopamine, Prussian blue, carbon, copper sulfide (CuS), bismuth sulfide (Bi_2_S_3_), and gold (Au) nanoparticles have been widely utilized to fabricate photothermal responsive DDSs.

Photodynamic responsive DDSs rely on photosensitizer-mediated PDT to allow NIR triggered drug release (Fig. 1) [45]. PDT utilizes photosensitizers and light irradiation to generate ROS to induce cancer cell death [46]. Meanwhile, the hypoxic condition of tumors will be aggravated due to the continuous consumption of oxygen molecules in PDT process [47]. In view of this, integrations of ROS- and hypoxia-sensitive moieties into DDSs enable the development of ROS- and hypoxia-responsive DDSs, respectively [31]. The common photosensitizers for PDT include tetraphenylchlorin (TPC), indocyanine green (ICG), Rose bengal (RB), chlorin e6 (Ce6), porphyrin, pheophorbide A (PhA), boron dipyrromethene (BODIPY), conjugated polymer (CP), and semiconducting polymer (SP). Among them, ICG has been approved by the US Food and Drug Administration (FDA) for the clinical diagnosis and phototherapy [31].

Photoconversion responsive DDSs are fabricated through integrating UV/visible light-sensitive components (such as 2-nitrobenzyl, spiropyran, coumarin, 7-nitroindoli, donor–acceptor Stenhouse adduct, and azobenzene) into upconverting nanosystems with loadings or conjugations of drugs [32]. Under NIR laser irradiation, upconversion materials convert NIR light into UV or visible light that can destroy these sensitive moieties [48], allowing for on-demand drug release (Fig. 1). The major upconversion materials used in such a type of DDSs are upconverting nanoparticles (UCNPs).

## Photothermal responsive DDSs

Due to the existence of thermal-responsive materials that exhibit conspicuous changes of their physical properties with temperature [5], a wide variety of photothermal responsive DDSs have been developed for cancer therapy. Based on the structures and components of nanocarriers, photothermal responsive DDSs mainly consist of photothermal responsive liposomes and photothermal responsive phase change material (PCM)-based hollow nanostructures [49–51].

### Photothermal responsive liposomes

Thermal responsive liposomes transformed from natural and/or synthetic fatty acids have been widely used to synthesize DDSs, especially for photothermal responsive DDSs [52–54]. Xia’s group synthesized a photothermal responsive liposome consisting of an eutectic mixture of natural fatty acids, a chemotherapeutic drug, doxorubicin (DOX) and a NIR dye (IR780) for NIR-triggered drug release [55]. Biocompatible PCM of eutectic mixture with a well-defined melt point at 39 °C was constructed using lauric acid and stearic acid. IR780 served as a photothermal agent to increase the temperature under 808 nm NIR laser irradiation, inducing the melt of liposomes for drug release. The cell death of human lung A549 cancer cells after treatment with DOX/IR780 DDSs upon NIR laser irradiation reached to 90%, which was 1.34-fold higher than that without laser irradiation, verifying the photothermal-controlled chemotherapeutic effect.

Soon afterwards, photothermal responsive liposomes with photocontrolled drug release profiles have been extensively developed and used for synergetic PTT/chemotherapy [56]. For example, a thermosensitive liposome was designed to contain CuS, DOX, and a NIR dye (MBA) encapsulated with stearic acid and lauric acid acting as the PCM [57]. The formed thermosensitive liposome (CuS-DOX-MBA@PCM) showed a relatively low eutectic point close to the body normal temperature. MBA and DOX could be released from this DDS upon 808 nm laser irradiation, while negligible MBA and DOX releases were observed without laser irradiation. As such, CuS-DOX-MBA@PCM afforded a high efficacy in killing A549 cancer cells and inhibiting growth of ehrlich ascites carcinoma (EAC) tumors in living mice after NIR-triggered drug release. In another study, Wang and Zhao et al. developed a thermosensitive liposome bilayers coated mesoporous carbon nanoparticle (MCN) to achieve NIR light triggered on-demand release of DOX for synergistic cancer PTT-chemotherapy [58]. 1,2-Distearoyl-sn-glycero-3-phosphoethanolamine-*N*-[amino(poly(ethylene glycol))] (DSPE-PEG), 1-stearoyl-2-hydroxy-sn-glycero-3-phosphatidylcholine (MSPC), and 1,2-dipalmitoyl-sn-glycero-3-phosphatidylcholine (DPPC) were used to construct surface liposome bilayers with a phase transition temperature (T_m_) of 40.7 °C. The release of DOX from DOX/MCN upon 808 nm laser irradiation was sharply increased compared to that without laser irradiation. After treatment with DOX/MCN and NIR laser irradiation, the growth of murine breast 4T1 tumors in living mice was much slower than that of mice after other treatments, indicating superior therapeutic effect of synergistic PTT/chemotherapy.

**Fig. 2:**
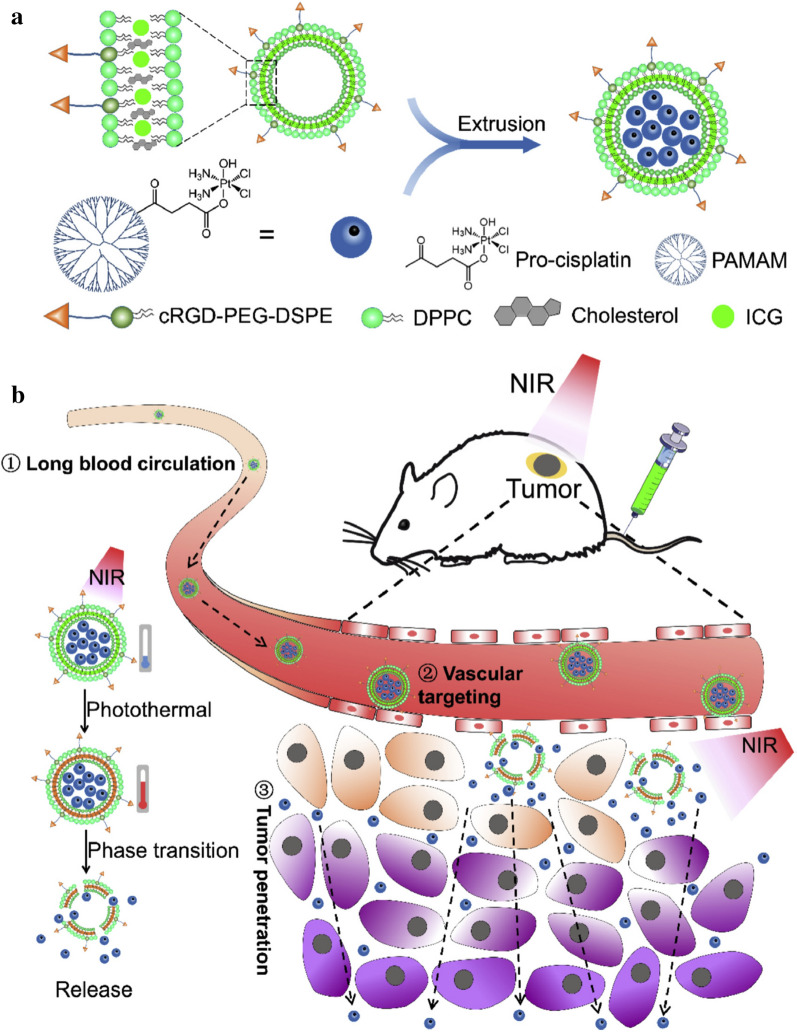
**a** Schematic illustration of formation of degradable liposomal PAM/Pt@IcLipo DDSs. **b** Schematic illustration of PAM/Pt@IcLipo DDSs for tumor vessel targeting, NIR light-triggered drug release and deep penetration in tumor tissues: (1) extending the circulation time of PAM/Pt in the blood; (2) targeting the tumor blood vessels; (3) being triggered to release PAM/Pt nanoparticles with a size less than 10 nm through NIR laser irradiation (Reproduced from Ref. [59] with permission from Elsevier, copyright 2020)

Photothermal responsive liposomes can also achieve improved penetration of drugs into deep tumors for further enhanced therapeutic efficacy. As demonstrated in a recent study of Zhou’s group [59], a degradable photothermal responsive liposome was constructed via a nanoprecipitation of cyclic arginine-glycine-aspartic acid (cRGD)-conjugated DSPE-PEG, DPPC, cholesterol, a photosensitizer (ICG), and poly(amidoamine) (PAMAM) dendrimers grafting cisplatin prodrug (PAM/Pt) (Fig. 2a). ICG was loaded in the out layer and PAM/Pt was encapsulated inside the formed liposome (PAM/Pt@IcLipo). PAM/Pt@IcLipo showed a long blood circulation time and an excellent targeting capability to tumors via cRGD-mediated targeting pathway. Under NIR laser irradiation at 808 nm, the photothermal effect of ICG raised the local temperature, which resulted in the destruction of PAM/Pt@IcLipo and release of tiny PAM/Pt (~ 8.6 nm). These tiny PAM/Pt could penetrate into deep tumor tissues to exert chemotherapy (Fig. 2b). Because of the synergistic action of PTT/chemotherapy and improved drug penetration depth, the highest therapeutic efficacy (91.1%) in suppressing the growth of 4T1 tumors was achieved through PAM/Pt@IcLipo injection plus 808 nm laser irradiation.

To further increase the therapeutic effect, several photothermal responsive liposomes have been developed for multimodal cancer therapy. As an example, a photothermal responsive liposome with loadings of ICG and DOX and conjugation of gadolinium chelates was synthesized by Fan and coworkers for imaging-guided PTT/PDT/chemotherapy [60]. The PCM core consisted of auric acid and stearic acid at a weight ratio of 4:1 showed a sharp melting point at 39 °C. Under NIR laser irradiation at 808 nm, ICG mediated photothermal effect and increased temperature to melt the PCM core, which enabled on-demand release of DOX. In contrast, the drug release from this liposome was negligible without laser irradiation. In addition, ICG exerted PDT to produce ROS under NIR laser irradiation. As a result, this liposome upon NIR laser irradiation mediated a synergistic PTT/PDT/chemotherapy, greatly inhibiting the growth of human cervical HeLa tumors in living nude mice. Similarly, Cui’s group developed a mitochondria-targeting photothermal responsive liposome via self-assembly of triphenylphosphine (TPP)-coupled DSPE-PEG, DPPC, 1,2-distearoyl-sn-glycero-3-phosphocholine (DSPC), cholesterol, a photosensitizer (IR-780) and lonidamine (LON) [61]. TPP-binding improved the internalization of the liposome into mitochondria of cancer cells. Laser irradiation at 808 nm triggered IR-780 to elevate the local temperature, which could be utilized for PTT and induced the release of LON from the thermosensitive liposome for chemotherapy. Meanwhile, IR-780 generated ROS in mitochondria for PDT to increase therapeutic efficiency. Such liposome-mediated synergetic PTT/PDT/chemotherapy led to complete eradication of murine Lewis lung LL/2 tumors in nude mice without recurrence within 50 days. In another study, an “all-in-one” photothermal responsive liposome was developed based on a small-molecule dye (DPP-BT) for second NIR fluorescence and photoacoustic dual-modal imaging guided synergetic PTT/PDT/chemotherapy [62]. DPP-BT and DOX were co-encapsulated into organic PCM core of lauric acid and stearic acid (eutectic point at 39 °C) with an out liposome layer of folic acid-modified DSPE-PEG and lecithin. DPP-BT not only served as a dual-modal contrast agent for imaging, but also acted as a therapeutic agent for PTT and PDT. Under NIR laser irradiation at 730 nm, DOX was effectively released from the formed P(DPP-BT/DOX) nanoparticles through NIR-induced PTT of DPP-BT. Upon accumulation of P(DPP-BT/DOX) into tumors of HeLa tumor-bearing mice, the temperature of tumor regions increased under laser irradiation and reached around 54 °C within 4 min, which was enough for PTT effect and to induce the melt of PCM matrix for DOX release. Both in vitro and in vivo results showed that such a liposome resulted in a remarkable antitumor efficacy via synergetic PTT/PDT/chemotherapy triggered by a single NIR laser irradiation.

**Fig. 3:**
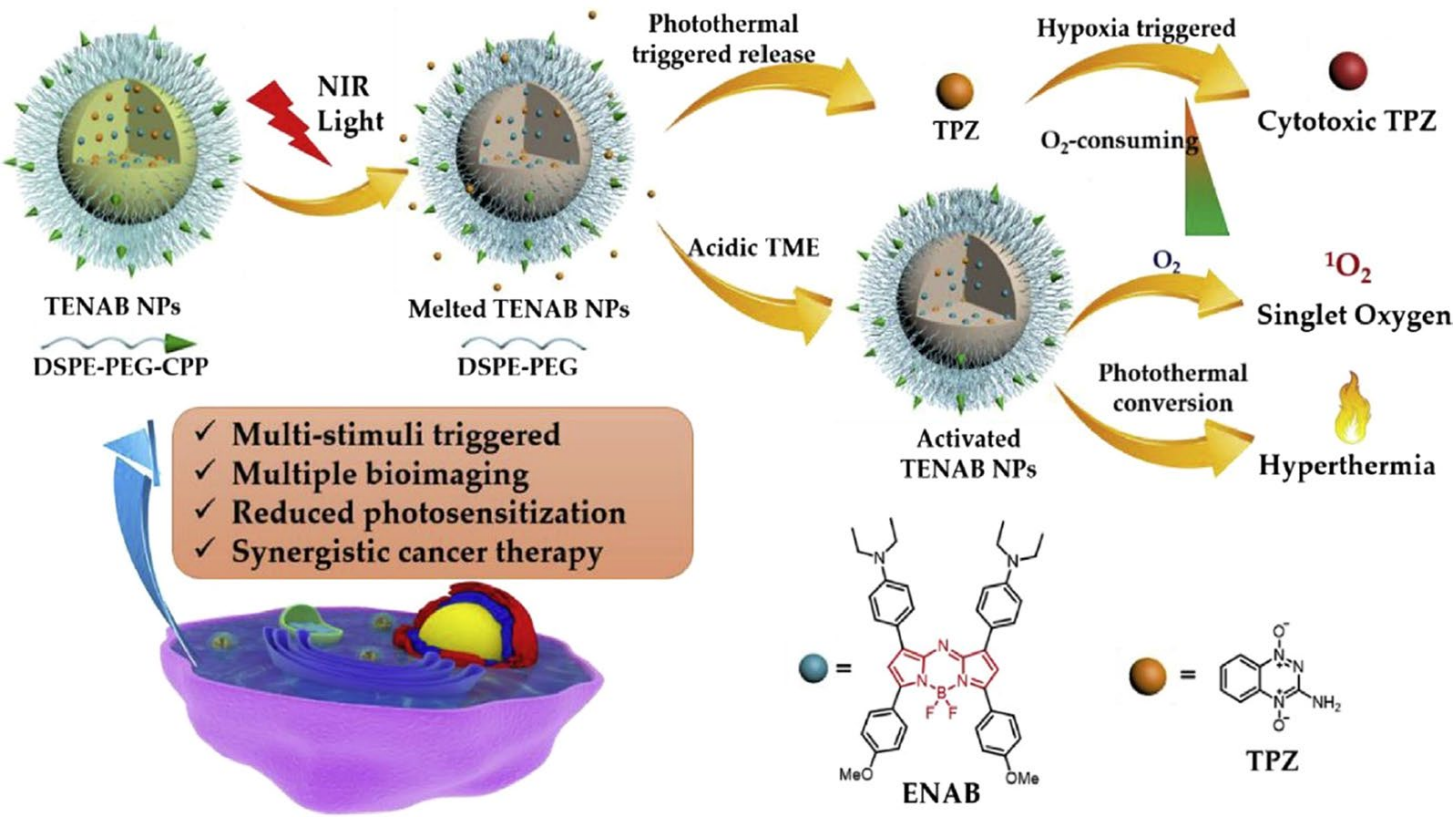
Schematic illustration of photothermal-pH-hypoxia multi-responsive TENAB nanoparticle for synergistic therapy (Reproduced from Ref. [63] with permission from Elsevier, copyright 2019)

Different from above photothermal responsive liposomes, Dong’s group recently developed a photothermal-pH-hypoxia multi-responsive liposome for synergistic cancer PTT/PDT/chemotherapy with negligible skin phototoxicity [63]. Such a multi-stimuli responsive liposome consisted of an eutectic PCM mixture of linoleic acid and stearyl alcohol with a melting point at around 43.9 °C, cell-penetrating peptide (CPP, YGRKKRRQRRR) modified DSPE-PEG, DSPE-PEG, a diethylamino group-flanked aza-BODIPY derivative (ENAB) as the pH-responsive photosensitizer, and a hypoxia-specific prodrug, tirapazamine (TPZ). Under acidic pH in tumor microenvironment, ENAB within the formed TENAB nanoparticle turned “off” its charge-transfer state, generating ROS for PDT and heat for PTT upon NIR laser irradiation at 808 nm. Meanwhile, the generated heat melted the PCM coating for release of TPZ (Fig. 3), while negligible release of TPZ was observed without laser irradiation. Subsequently, in the hypoxic condition aggravated by PDT, TPZ was activated into cytotoxic form for chemotherapy. As a result, TENAB nanoparticle afforded a synergistic action of PTT/PDT/chemotherapy upon NIR laser irradiation, completely eradicating HeLa tumors in living nude mice.

Via loading anticancer drugs and genes, photothermal responsive liposomes can be developed for synergistic PTT/chemo-/gene therapy. For example, Nie’s group synthesized a thermo-sensitive co-polymer, poly(2-(2-methoxyethoxy) ethyl methacrylate-*co*-oligo (ethylene glycol) methacrylate)-*co*-2-(dimethylamino) ethyl methacrylate-*b*-poly(d,ʟ-lactide-*co*-glycolic acid) and used it to construct a liposome with co-encapsulations of DOX and paclitaxel (PTX) and absorption of small interfering RNAs against surviving [64]. The liposome was surface coated with polydopamine, which conferred it with photothermal effect and also protected the burst release of drugs. Under NIR laser irradiation at 808 nm, polydopamine generated heat via PTT effect, resulting in collapse of nanoparticles and subsequent release of drugs within tumors. Such a synergistic action of PTT/chemo-/gene therapy resulted in complete regression of human breast MDA-MB-231 tumors, with a decrease in the chemotherapeutic drug dosage to about 1/20 of conventional dose.

Apart from phase transitions of PCM, the controlled drug release from photothermal responsive liposomes can also be achieved via photothermal-mediated generation of gas. For example, Liang’s group fabricated a thermoresponsive cerasome-forming liposome via self-assembly of a NIR agent, 1,1′-dioctadecyl-3,3,3′,3′-tetramethylindotricarbocyanine iodide (DiR), cerasome-forming lipid, DSPE-PEG, a hyperthermia-responsive ammonium bicarbonate (ABC) and DOX for NIR light-triggered drug release and synergistic cancer PTT/chemotherapy [65]. Due to the good stability of cerasome, both DOX and DiR retained in DDSs at body temperature without obvious releases during blood circulation. Under NIR laser irradiation at 760 nm, DiR-mediated photothermal effect to increase temperature, resulting in the degradation of ABC to generate CO_2_ bubbles. The generated CO_2_ bubbles increased the permeability of cerasome bilayers to allow DOX release. As such, a high therapeutic effect in eradicating tumors of living mice was achieved via an intravenous injection of DOX/DiR/ABC cerasome liposome followed by laser irradiation of tumors. In another study, Du and coworkers constructed a dual aptamer-modified and gold nanoshell coated thermosensitive liposome with encapsulation of ABC in the internal aqueous phase and an anti-cancer drug, docetaxel (DTX) in the phospholipid layer [66]. Similarly, gold nanoshell-mediated photothermal effect under 808 nm laser irradiation induced the generation of CO_2_ bubbles, which increased the permeability of bilayers for drug release. In addition to PTT and chemotherapy, the AS1411 aptamer that could dispute the functions of nucleolin via binding to nucleolin in nuclear membrane was used as a biotherapeutic agent. As a result, this DDS showed a high therapeutic efficacy in suppressing the growth of murine sarcoma S180 tumors via synergistic PTT/chemotherapy/biological therapy.

### Photothermal responsive PCM-based hollow nanostructures

The combinations of hollow nanostructures and PCM can be utilized to construct photothermal responsive hollow DDSs [67]. The photothermal effect of optical materials in nanocarriers elevates local temperatures, which induces the phase transition of PCM from a solid to a liquid, leading to precise release of drugs from hollow cavities.

Mesoporous silica nanoparticles (MSNs) have been used to develop photothermal responsive DDSs for cancer therapy [68–70]. For example, Kim and Yong et al. constructed a mesoporous silica coated silver–gold hollow nanoplatform to precisely regulate the release of 5-fluorouracil (anticancer drug) for prostate cancer therapy and photothermal therapy [70]. The mesopores were capped with a thermosensitive PCM (lauric acid), which allowed for remote, precise, and spatiotemporal control of drug release via silver–gold nanoshell-mediated photothermal heating under NIR laser irradiation at 808 nm. Such a nanoplatform thus showed a synergistic effect in killing cancer cells. Since MSNs do not have photothermal effect, other photothermal agents are required to construct these MSN-based photothermal responsive DDSs.

**Fig. 4:**
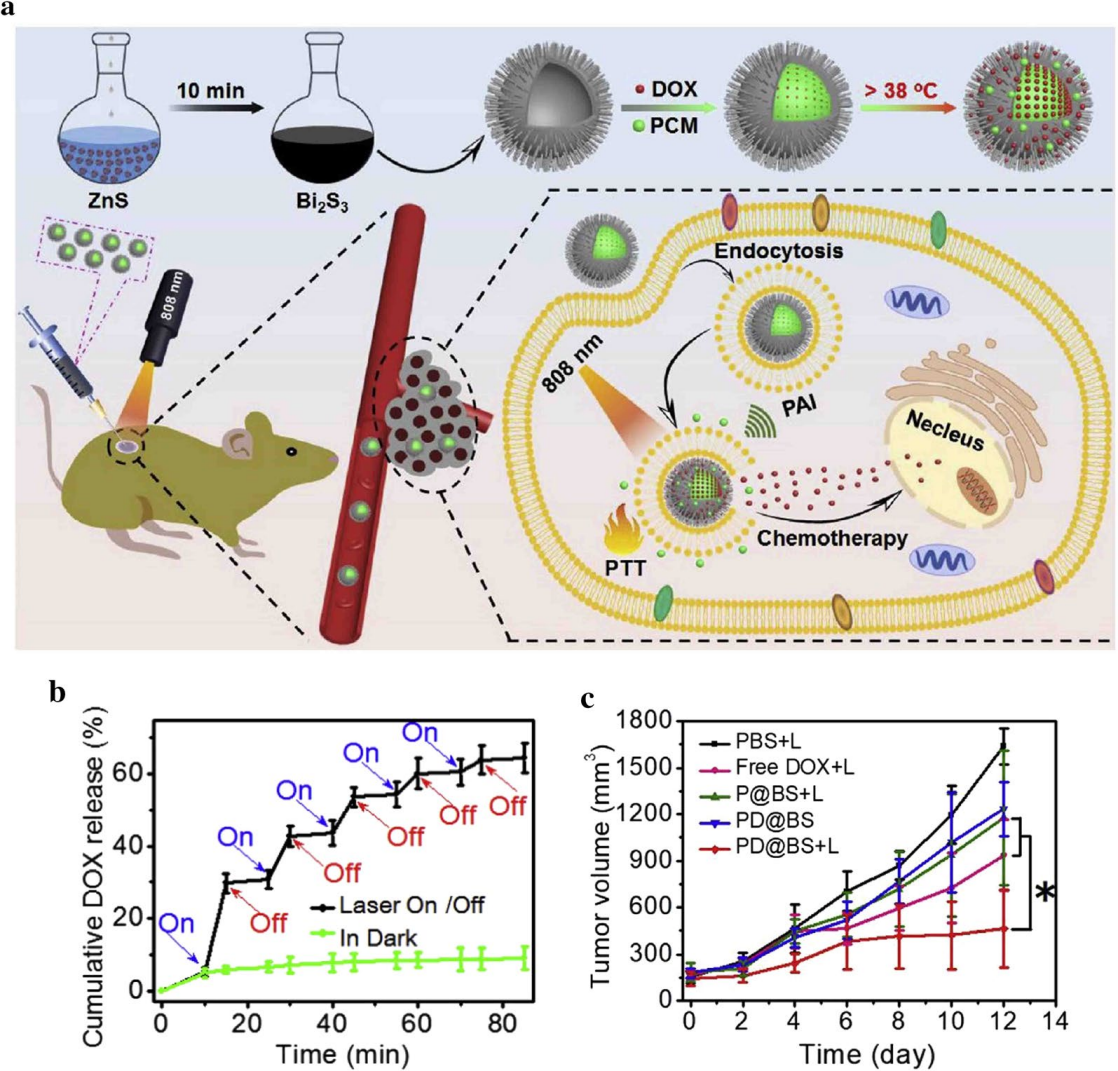
**a** Schematic illustration of thermosensitive urchin-like Bi_2_S_3_ hollow nanoparticles as photothermal responsive DDSs for photothermal-chemotherapy. **b** Cumulative release of DOX from PD@BS induced by cyclic “turn-on” and “turn-off” of NIR light at different times. **c** The growth curves of MDA-MB-231 tumors in different groups during a period of 12 days (Reproduced from Ref. [71] with permission from Elsevier, copyright 2020)

Qian’s group developed a photothermal responsive DDS based on rod-based urchin-like Bi_2_S_3_ hollow nanoparticles (termed as U-BSHM) to allow precise release of chemotherapeutic agents for synergistic PTT/chemotherapy [71]. A sacrificial template engaged polyol route was used to synthesize U-BSHM as the photothermal agent with a photothermal conversion efficiency of 26.8%. U-BSHM was loaded with DOX and encapsulated with PCM of 1-tetradecanol (the melting point at around 38 °C) as the “gatekeeper” to form the photothermal responsive DDS (termed as PD@BS) (Fig. 4a). The release of DOX was rapidly increased upon NIR laser irradiation at 808 nm compared to that without laser irradiation (Fig. 4b). The cell viability of MDA-MB-231 cancer cells gradually decreased with the prolonged NIR laser irradiation time and elevated concentrations of DDSs, which was due to the synergetic action of PTT and released DOX induced by NIR laser irradiation. The temperature of MDA-MB-231 tumor regions of living mice after intratumoral injection of PD@BS gradually increased to a plateau of around 49 °C under NIR laser irradiation, which was enough to induce the release of DOX and ablate tumors. As a result, the therapeutic effect of PD@BS was obviously improved with NIR laser irradiation (Fig. 4c).

Several other photothermal agents have also been used to induce the phase changes of PCM to achieve on-demand releases of chemotherapeutic drugs and synergistic effects of PTT/chemotherapy. Cai and co-workers constructed a photothermal responsive DDS based on hollow magnetic Prussian blue nanoparticles for NIR light-triggered PTT/chemotherapy [72]. Such a DDS consisted of a Prussian blue coated hollow iron oxide magnetic nanoparticle as the carrier and photothermal agent, DOX as the chemotherapeutic drug, and a biocompatible PCM of 1-pentadecanol as the “drug-janitor” for controlled-release in response to increased temperature (> 42 °C). In such a system, the release of DOX from DDS was significantly increased upon 808 nm laser irradiation due to Prussian blue-mediated photothermal effect. After treatments, this system exerted synergistic PTT/chemotherapy and afforded an enhanced therapeutic efficacy in inhibiting the growth of human hepatoma HepG2 tumors compared to sole chemotherapy and PTT. Similarly, Guo’s group developed a mesoporous carbon nanoparticle (MCN)-based DDS filled with DOX and 1-tetradecano as the PCM in hollow cavities for NIR light-triggered release of DOX [73]. The cumulative DOX release was significantly increased under 808 nm laser irradiation via MCN-mediated photothermal effect. A much higher intracellular DOX level was observed in MCF-7/ADR cells after treatment with MCN-based DDS plus laser irradiation relative to those without laser irradiation. The apoptosis evaluation showed that the highest percentage of total apoptosis (49.7%) was caused by MCN-based DDS treatment plus NIR laser irradiation, which was 2.48-fold higher relative to that of free DOX treatment alone. This suggested an enhanced therapeutic effect induced by the NIR light-triggered PTT and drug release.

In addition to PTT/chemotherapy, photothermal responsive PCM-based hollow nanostructures have also been developed for synergistic PTT/PDT/chemotherapy. For example, hollow mesoporous ZrO_2_-coated Nd^3+^-doped UCNPs were used as the photothermal nanocarriers to load DOX, Ce6, and 1-tetradecanol (PCM), forming a DDS (termed as UCNPs@ZrO_2_-Ce6/DOX/PCM) [74]. Due to the UCNP-mediated PTT, the release efficiency upon 808 nm NIR laser irradiation reached 44.12%. The inhibitory effect of HeLa cancer cells after treatment of UCNPs@ZrO_2_-Ce6/DOX/PCM and laser irradiation reached nearly 100%, which was 3.2-, 2.3-, and 1.6-fold higher than that in sole PTT, free DOX and PTT + PDT group, respectively. An excellent in vivo synergistic antitumor efficacy was also verified in murine cervical U14 tumor-bearing mice after treatment with this nanosystem plus NIR laser irradiation. Similarly, Xu’s group constructed another photothermal responsive DDS (termed as HPDC) with a high photothermal conversion efficiency of 44.13% for synergistic PTT/PDT/chemotherapy [75]. Such a HPDC consisted of four key components: hollow mesoporous CuS nanoparticle as both the photothermal agent and nanocarrier, surface coated 1-tetradecanol as a PCM, DOX as a chemotherapy drug and Ce6 as a photodynamic photosensitizer. NIR laser irradiation at 808 nm increased local temperature via CuS-mediated PTT, which obviously increased the release of both DOX and Ce6. Their cumulative release reached 72.78 and 74.4% after six cycles of NIR laser irradiation, respectively, while only approximately 5% DOX or Ce6 in total was released without laser irradiation. Via HPDC-mediated synergistic PTT/PDT/chemotherapy, the growth of 4T1 tumors in living mice was almost completely inhibited, while other treatments failed to effectively suppress tumor growth.

This section summarizes the recent constructions of photothermal responsive liposomes and PCM-based hollow nanostructures for NIR photoactivated drug release through photothermal effect mediated phase transition of thermal-sensitive materials. The liposomes have intrinsic advantages of good biocompatibility and biodegradability, low toxicity and immunogenicity, tunable physicochemical and biophysical properties and unique capability of loading both lipophilic and hydrophilic drugs [76]. However, liposomes often exhibit low stability because the phospholipid can be easily oxidized and hydrolyzed. Moreover, the use of organic solvent or high temperature during the liposome fabrication process may affect the bioactivity of drug molecules [77]. The hollow nanostructure-based DDSs exhibit various advantages including excellent chemical stability, high drug loading capability, and abundant surface chemical groups for further functionalization [78–80]. However, they generally have the drawbacks of poor biodegradability and long-term toxic concerns in living bodies [81]. These disadvantages of photothermal responsive DDSs should be considered to facilitate their translation for clinical medicine.

## Photodynamic responsive DDSs

In the typical process of PDT, photosensitizers not only convert light energy into ROS, but also deplete oxygen to increase the tumor hypoxia to a certain extent [47]. Hence, it is feasible to construct photodynamic responsive DDSs via integrating ROS- or hypoxia-cleavable moieties into DDSs.

### ROS-responsive DDSs

ROS-responsive DDSs have been applied to selectively release various drugs into target tissues, which can be achieved via photo-controlled cleavage of ROS-responsive linkers [12, 82, 83]. For instance, Schnermann et al. reported the utilization of NIR light to cleave antibody–drug conjugates containing a cyanine photocage [84]. Such conjugates consisted of a heptamethine cyanine fluorophore serving as the photocaging component, combretastatin A4 (CA4) acting as the potent inhibitor of microtubule polymerization and a human epidermal growth factor receptor (EGFR)-binding monoclonal antibody. This linker strategy utilized carbamate functional groups as the antibody attachment points, which ensured the release of CA4 drugs from antibodies triggered by ROS generation form fluorophore under NIR laser irradiation at 690 nm. Moreover, the fluorescence signal of this system provided a useful marker to verify the accumulation of conjugates, while the loss of fluorescence signal after excitation by NIR light indicated drug release.

**Fig. 5:**
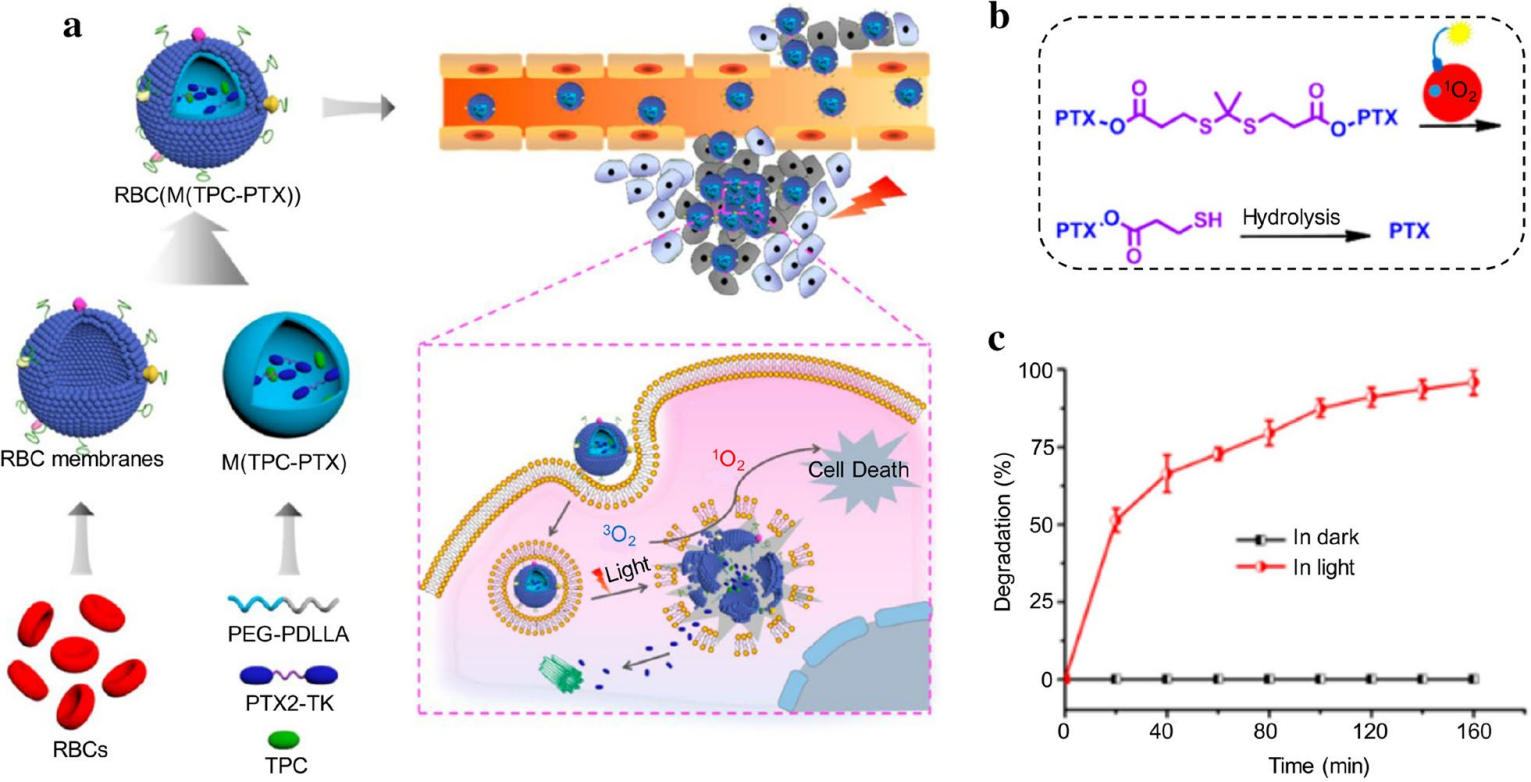
**a** Schematic illustration of synthesis and mechanism of RBC membrane-coated dimeric prodrug DDSs with NIR light triggered on-demand drug release for synergistic PDT/chemotherapy. **b** Schematic illustration of mechanism of ^1^O_2_ induced activation of PTX2-dithioketal prodrug. **c** The degradation of PTX2-dithioketal in DDSs over time upon irradiated by 638 nm laser lamp (100 mW/cm^2^) tested by high-performance liquid chromatography (HPLC) (Reproduced from Ref. [86] with permission from American Chemical Society, copyright 2018)

Another representative example of ROS-responsive DDS was demonstrated by Liu’ s group [85]. In this system, DOX was covalently conjugated to an organic conjugated polyelectrolyte (CPE) through a ROS-cleavable dithioketal linker. PEG chains and cRGD were also conjugated to the backbone of CPE to improve its solubility and target specificity to cancer cells, respectively. CPE was utilized as the photosensitizer to generate ROS upon white light irradiation, not only exerting PDT, but also triggering the cleavage of dithioketal linkers for on-demand DOX release, which permitted a synergistic cancer therapy with an enhanced therapeutic effect. The ROS-cleavable dithioketal linker was also utilized by Xie’s group to construct a photoactivatable biomimetic dimeric prodrug DDS with red blood cell (RBC) membrane camouflage for synergistic cancer PDT/chemotherapy (Fig. 5a, b) [86]. It has been demonstrated that cell membrane camouflaged nanomaterials exhibit a prolonged blood circulation, and tumor targeting ability, thus enabling an enhanced tumor accumulation and improved therapeutic outcomes [87–90]. After the effective cellular internalization of biomimetic prodrug DDS by cancer cells, the photosensitizer, tetraphenylchlorin (TPC) embedded in the inner core could generate ROS upon NIR light irradiation at 638 nm, leading to synergetic therapy through photodynamic disruption of the cellular endosomes, and meanwhile, cleavage of the dithioketal linkers to amplify the release of PTX (Fig. 5c). The therapeutic effect was significantly increased via such a biomimetic prodrug DDS-mediated synergistic PDT/chemotherapy.

In addition, a singlet oxygen (^1^O_2_) sensitive bis-(alkylthio)alkene (BATA) linker has been used to construct ROS-responsive DDSs to achieve on-demand chemotherapy upon irradiation of light [91]. An interesting example was demonstrated by Liu and coworkers [92]. In their system, photosensitizer (Ce6) doped mesoporous silica nanorods (CMSNRs) were coated with bovine serum albumin (BSA) via the BATA linkers and then modified with PEG, forming a drug delivery nanocarrier that was able to load small drug molecules such as DOX, or larger cargos such as *cis*-Pt(IV) prodrug conjugated third generation dendrimer (G3-Pt). Upon NIR laser irradiation at 660 nm with a low power density (down to 5–50 mW/cm^2^), the doped Ce6 generated ^1^O_2_ to effectively cleave the BATA linkers, which induced detachments of BSA-PEG from the surface of nanocarriers and thus triggered a release of loaded DOX or G3-Pt. As a result, both the in vitro and in vivo therapeutic effects were enhanced through the on-demand release of therapeutic cargos under NIR light irradiation.

Diselenide bond (Se–Se) that can be cleaved by ROS has shown to be another good candidate as light-sensitive linker for the construction of ROS-responsive DDSs [93]. Xu and colleagues developed a light‐responsive micelle with co-encapsulation of photosensitizer (porphyrin) and DOX based on the diselenide bond [94]. The ^1^O_2_ generated from porphyrin under light (600–780 nm) irradiation facilitated the cleavage of diselenide bonds, resulting in disruption of micelles and on-demand release of DOX. Similarly, Yang’s group developed a light‐responsive poly(methacrylic acid) (PMAA)-based nanogel system based on diselenide-cross-linkers [95]. ICG was loaded into nanogels to generate ^1^O_2_ upon NIR laser irradiation at 785 nm, which induced disassembly of diselenide‐crosslinked nanogels and thus achieved on-demand release of loaded DOX.

In another study, a ROS-responsive DDS was constructed via self-assembly of PEG-stearamine (C18) conjugate with the ROS-cleavable thioketal linkers and co-loaded DOX and a photosensitizer pheophorbide A (PhA) for enhanced cancer PDT/chemotherapy [96]. Upon NIR laser irradiation at 670 nm, the formed DDS (termed as PTS-DP) generated ^1^O_2_ due to the photodynamic effect of PhA, and the generated ^1^O_2_ cleaved thioketal linkers in polymer conjugates, leading to rapid dissociation of DDSs for DOX release. The gradual elevation of local ROS levels mediated by PDT synergized with NIR light-triggered release of DOX, showing an enhanced efficacy to treat murine colorectal CT26 tumors.

In addition, the ROS sensitivity of aminoacrylate groups was used to develop a ROS-responsive supramolecular DDS with an optimized loading ratio of the photosensitizers and prodrugs for cancer therapy [97]. A diblock copolymer was synthesized to consist of PEG, poly-ʟ-glutamic acid (PGA) and β-cyclodextrin (β-CD). Adamantane-conjugated aza-BODIPY (Ada-BODIPY) and PTX (Ada-PTX) were used as the photosensitizer and prodrug guest molecules, respectively. Via a strong interaction between β-CD and the adamantane units, the supramolecular DDS was formed. Under 660 nm laser irradiation, the photosensitizers generated ROS for PDT, and meanwhile to cleave the ROS-sensitive aminoacrylate groups in Ada-PTX to allow PTX release. Hence, the supramolecular DDS enabled a synergistic action of PDT/chemotherapy for inhibition of HeLa tumor growth.

### Hypoxia-responsive DDSs

**Fig. 6:**
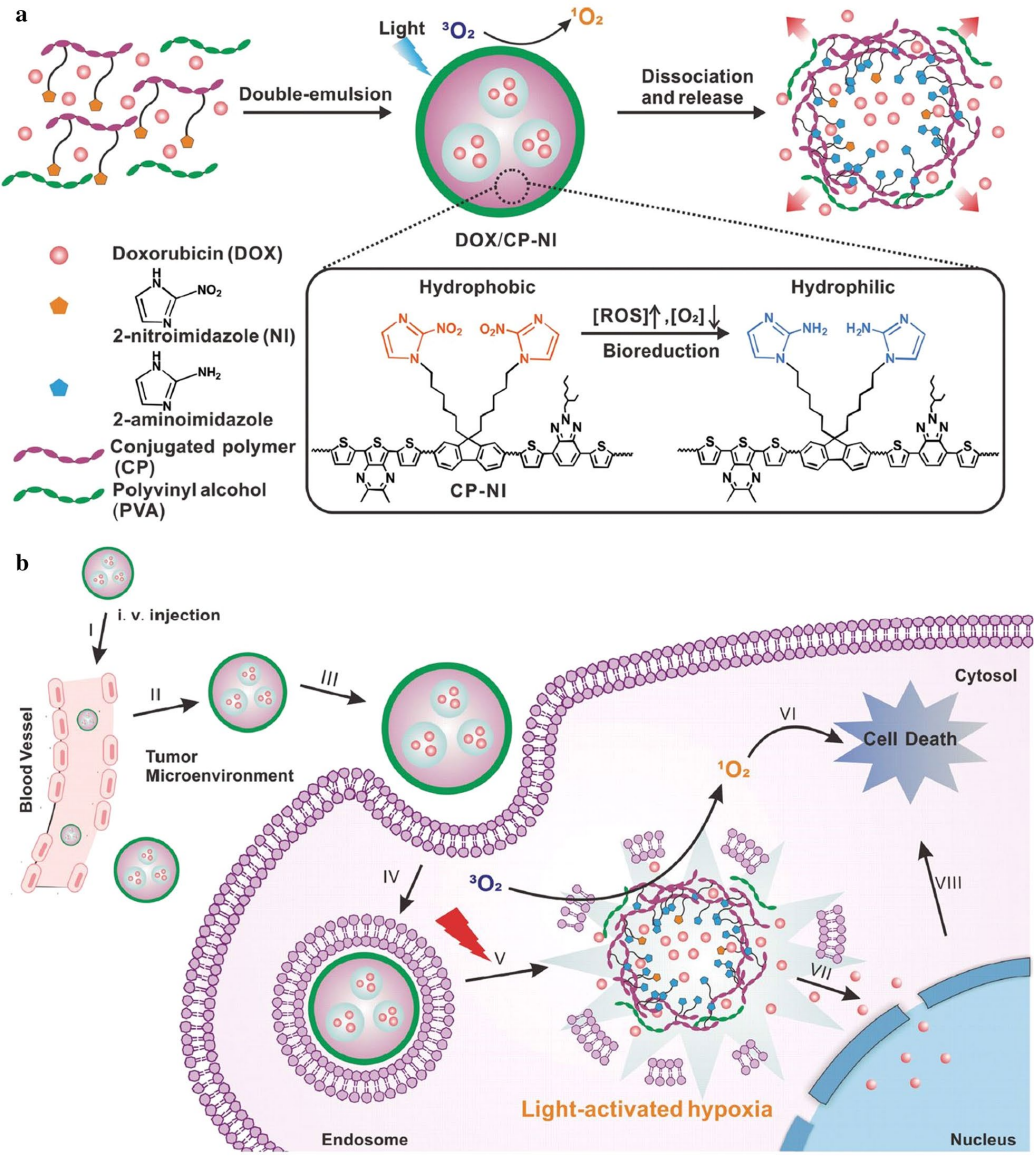
**a** Schematic of the formation and mechanism of light-activated hypoxia-responsive DOX/CP-NI. **b** Schematic of DOX/CP-NI mediated generation of ROS and induction of a local hypoxic environment for hypoxia-responsive release of DOX into cell nucleus for synergistic anticancer efficacy (Reproduced from Ref. [98] with permission from Wiley-VCH, copyright 2016)

In view of aggravated hypoxia in tumor microenvironment after PDT process, hypoxia-sensitive moieties have been used to construct hypoxia-responsive DDSs for synergistic PDT/chemotherapy. For example, Gu’s group constructed a novel DDS to realize NIR light triggered ROS generation and subsequent hypoxia-activated drug release [98]. This DDS was constructed via using a ROS-generating and hypoxia-responsive 2-nitroimidazole-grafted conjugated polymer (CP-NI) to encapsulate DOX through a double-emulsion-based solvent evaporation/extraction method (Fig. 6a). Upon 635 nm laser irradiation, the formed DDS (termed as DOX/CP-NI) generated ^1^O_2_ to permit PDT. Meanwhile, the dissolved oxygen was rapidly consumed due to the generation of ^1^O_2_, leading to a local hypoxic microenvironment. As a result, hydrophobic 2-nitroimidazole groups in the CP-NI were converted to hydrophilic 2-aminoimidazoles under the hypoxic condition, leading to the dissociation of DDS, and subsequent release of DOX. As expected, about 60% of DOX was released from the DOX/CP-NI at pH 7.4 after laser irradiation, but the released amount was very low without laser irradiation. Such a light-activated and hypoxia-responsive nanocarrier exerted synergistic PDT/chemotherapy, resulting in complete inhibition of the growth of HeLa tumors (Fig. 6b). In another similar study, 2-nitroimidazole derivative conjugated PEG amphoteric polymer-based DDSs with loadings of Ce6, gene probe, and a hypoxia-activated prodrug TPZ were fabricated through self-assembly for synergistic PDT/chemotherapy [99]. Upon NIR laser irradiation at 670 nm, Ce6-mediated PDT induced a hypoxic condition, which resulted in disassembly of DDSs for drug release and also activated the antitumor effect of TPZ for improved therapeutic effect. In vivo studies verified the greatly improved anti-cancer activity of such a DDS-mediated synergistic PDT/chemotherapy in inhibiting human breast MCF-7 tumor growth compared to sole PDT.

In another study, Pu’s group constructed a semiconducting polymer nanoparticles (SPNs)-based DDS for hypoxia-activated photodynamic cancer therapy [100]. The SPN-based DDS was formed via a self-assembly of the amphiphilic semiconducting brush polymers that comprised a photodynamic SP grafted with PEG and conjugated with a chemotherapeutic drug (bromoisophosphoramide mustard intermediate, IPM-Br) through a hypoxia-cleavable linker. Upon NIR laser irradiation at 808 nm, the SPN core generated ^1^O_2_ for PDT, and meanwhile, depleted oxygen to further promote tumor hypoxia. The hypoxia-cleavable linker was then cleaved by hypoxia and thus on-demand release of IPM-Br was achieved. After systemic administration, this DDS effectively accumulated at the tumor of living mice due to their small size and stealthy PEG grafting. Under NIR laser irradiation, the SPN-based DDS exerted synergistic PDT/chemotherapy and effectively inhibited the growth of 4T1 tumors.

The recent development of photodynamic responsive DDSs for PDT-synergistic chemotherapy has been introduced in this section. Although controllable drug releases from nanocarriers have been demonstrated, photodynamic responsive DDSs still have some unsatisfied drawbacks that need to be overcome. These DDSs can also respond to endogenous ROS or hypoxia in the biological systems, which may result in off-target drug release and thus cause side effects. In addition, the majority of photosensitizers in these systems are small molecular organic dyes that display poor optical stability upon light exposure [101]. Therefore, development of photosensitizers with good photostability will be a key step to realize wide applications of photodynamic responsive DDSs.

## Photoconversion responsive DDSs

Photosensitive moieties, such as 2-nitrobenzyl, coumarin, 7-nitroindoli, and azobenzene have been widely used to construct DDSs, whereas the majority of these moieties typically respond to short-wavelength deep-blue or UV light [102–105], which greatly limits in vivo applications of these DDSs. To address this issue, upconverting materials, such as UCNPs can be integrated into DDSs to convert NIR light into short-wavelength light sources [106].

Photoconversion-induced drug release from DDSs can be achieved via photolysis strategy. Li and colleagues encapsulated an 7-amino-coumarin derivative caged chlorambucil prodrug (termed as ACCh) into yolk–shell structured nanocarriers with UCNPs as moveable core and mesoporous silica as shell [107]. Under NIR laser irradiation at 980 nm, the UCNP core upconverted NIR light into UV emission (365 nm), effectively triggering the photolysis of ACCh prodrug to release chlorambucil, which facilitated the diffusion of activated drugs from nanocarriers through pores in the silica shell. This photoconversion responsive DDS afforded a high therapeutic efficacy in inhibiting the growth of S180 tumors and increasing survival of tumor-bearing living mice. In another study, Han’s group reported an organic-chromophore-based triplet–triplet annihilation upconversion (TTA-UC) strategy for NIR light-triggered cancer chemotherapy [108]. The developed DDSs consisted of MSNs loaded with TTA-UC molecules as the core, and an amphiphilic polymer encapsulated coumarin-chlorambucil prodrugs as the shell. Upon laser irradiation at 650 nm, TTA-UC molecules upconverted far-red light into blue emission to activate the prodrugs via photolysis. More than 48% activation of the prodrugs was achieved within 30 min, and a maximum photorelease of around 82% of prodrugs after 60 min of laser irradiation. As a result, the TTA-UC-based DDS mediated drug release allowed effective inhibition of 4T1 tumor growth in living mice.

UCNP-mediated NIR-to-UV conversion can also be utilized for photoreduction of platinum(IV) (Pt(IV)) prodrugs to form toxic Pt(II) [109]. A representative example was presented by Lin and coworkers, in which, photoconversion responsive DDSs were constructed via conjugating UCNPs with Pt(IV) prodrugs and a monolayer of PEG [110]. Under 980 nm NIR laser irradiation, UCNPs excited UV light to activate Pt(IV) prodrug into Pt(II) drug. More interestingly, the Pt(IV) prodrug-conjugated UCNPs under 980 nm laser irradiation showed a high efficiency in inhibiting the growth of murine hepatoma H22 tumors than that under direct UV light irradiation, although UV light could also effectively activate the Pt(IV) prodrugs. Similarly, Xing’s group developed a photoconversion responsive DDSs via conjugating UCNPs with Pt(IV) prodrugs and a short peptide probe [111]. Upon NIR light irradiation at 980 nm, the converted emission from UCNPs locally activated the Pt(IV) prodrug and thus efficiently induced a potent antitumor efficacy.

In a recent study, a Pt(IV) prodrug based charge-convertible DDS with the loading of NaYF_4_:Yb,Tm UCNPs and surface coating of a layer anionic PEG-poly(allylamine hydrochloride)-dimethyl-maleic acid polymer was developed for cancer therapy [112]. After response to the mild acidic stimulus (pH ~ 6.5) of tumor extracellular microenvironment, the anionic polymer underwent a charge-shifting to form a cationic polymer, leading to electrostatic repulsion and releases of positive Pt(IV)-UCNPs that could effectively bind to the negative cell membrane for cell internalization. Under 980 nm laser irradiation, UCNPs emitted UV light to efficiently activate the Pt(IV) prodrugs into highly cytotoxic Pt(II). Such a NIR photoresponsive smart DDS thus displayed a markedly enhanced tumor ablation efficacy in murine cervical U14 tumor-bearing Kunming mice.

In addition to photolysis and photoreduction, photoswitchable strategy has been used to develop photoconversion responsive DDSs. Shi’s group developed a photoconversion responsive DDS based on photoswitchable strategy to achieve NIR light-triggered release of anticancer drugs [113]. Mesoporous silica coated UCNPs were inside installed with “photomechanical” azobenzene (azo) groups acting as the “stirrer” and surface conjugated with a transactivator of transcription peptide to enable an enhanced cellular internalization into cancer cells, following by loading of DOX through formation of strong hydrogen bonds and charge interactions with the surface silanol groups. Upon NIR laser irradiation at 980 nm, UCNPs emitted UV and visible light, which triggered the switch of azo molecules between *trans* and *cis* isomer. Such a reversible photoisomerization created a continuous rotation-inversion movement, leading to the release of DOX from such a DDS. As a result, the DOX release percentage reached a maximum of 80% under intermittent NIR laser irradiation, while less than 5% of DOX was released without laser irradiation. This photoconversion responsive DDS was verified to show a high efficacy in killing cancer cells.

**Fig. 7:**
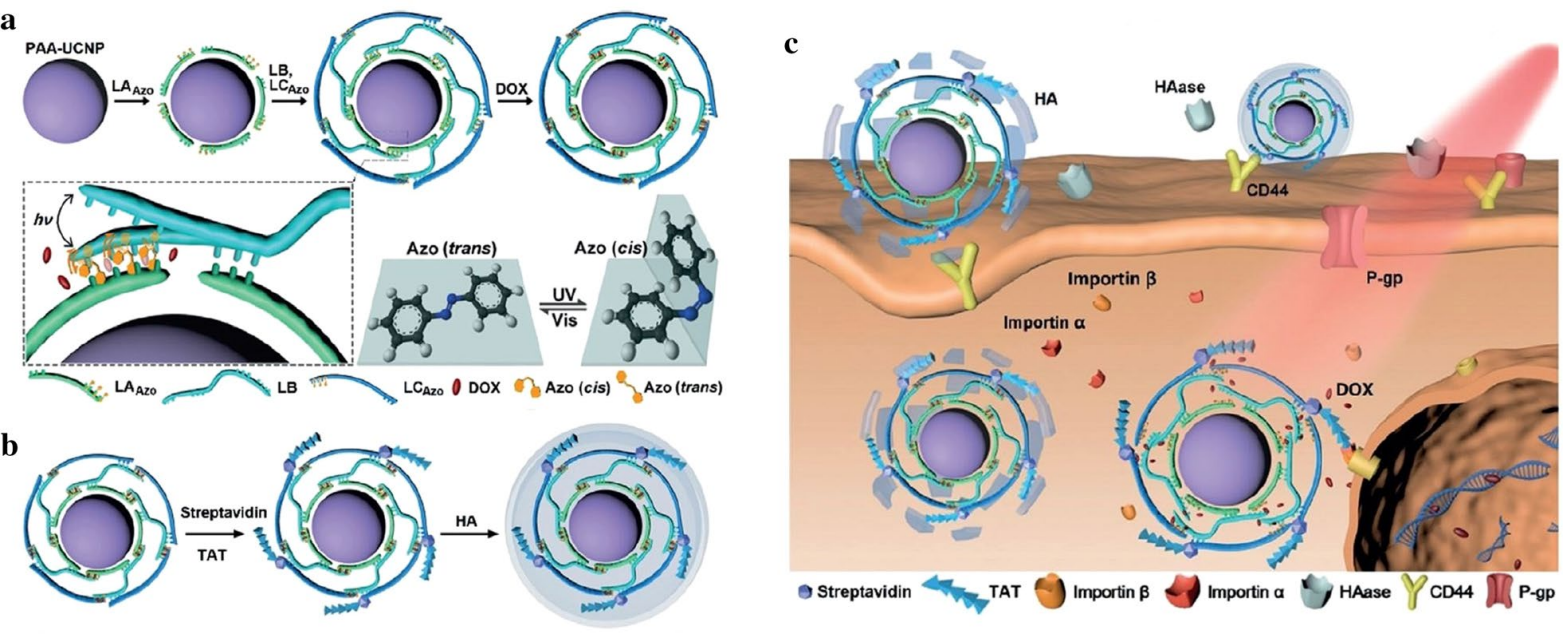
**a** Schematic illustration of assembly of UCNPs-LA_Azo_BC_Azo_/DOX. The enlarged section delineates the continuous photoisomerization of azo and cyclic hybridization and dehybridization of LA_Azo_ and LB (DNA strands LA_Azo_, LC_Azo_ with 3 azo moieties per DNA strand). **b** Synthesis of UCNPs/DOX-TAT-HA. **c** Illustration of HA-mediated endocytosis, TAT-mediated nuclear targeting and NIR-triggered drug release in living cells (Reproduced from Ref. [114] with permission from Wiley-VCH, copyright 2019)

Another azo-based DDS with controllable intracellular drug release upon NIR photoirradiation was constructed by Ju’s group for cancer therapy [114]. Such a DDS was constructed by assembling azo-functionalized DNA strands on poly(acrylic acid) (PAA)-modified UCNPs, followed by loading of DOX into the DNA helix (Fig. 7a). Under NIR laser irradiation at 980 nm, UCNPs emitted both UV and visible lights to fuel continuous photoisomerization of azo, which induced controllable DOX release due to the hybridization and dehybridization of cyclic DNA (Fig. 7b). The maximum DOX release amount reached 86.7% after 30 min of NIR laser irradiation. Through assembling a nuclear localizable HIV-1 trans-activator of transcription (TAT) peptide and hyaluronic acid (HA) onto the surface of DDS, targeting release of DOX inside cancer cell nucleus was achieved upon NIR laser irradiation (Fig. 7c). Monitoring of tumor growths after different treatments showed that this DDS (UCNPs/DOX-TAT-HA)-mediated therapy had a significantly improved chemotherapeutic outcome for HepG2 tumors in living mice.

A NIR photoswitchable cage mimicking DDS was developed through anchoring a photochromic spiropyran onto mesoporous silica coated UCNPs with loading of curcumin for cancer therapy [115]. The hydrophobic spiropyran formed a compact layer on silica shells to conceal curcumin inside the channels of nanocarrier without unexpected drug release. Upon NIR irradiation at 980 nm, UCNPs effectively converted NIR light to UV emission light that induced the conformational transformation of spiropyran molecules from hydrophobic to hydrophilic state. Such a NIR photoirradiation caused hydrophobicity-hydrophilicity switch achieved on-demand release of curcumin with good bioactivity for cancer chemotherapy. Moreover, the UV/visible light produced by UCNPs activated curcumin to initiate the generation of ROS, further improving the therapeutic efficiency. Such a photoconversion responsive DDS was demonstrated to exhibit a significantly enhanced antitumor efficiency in 4T1 tumor-bearing mice.

In another study, a multifunctional UCNP-based micelle with NIR photocontrolled drug release was developed for combinational cancer PDT and chemotherapy [116]. The micelle was formed via modifying UCNP with a photosensitive amphiphilic copolymers poly(4,5-dimethoxy-2-nitrobenzyl methacrylate)-PEG (PNBMA-PEG) and a photosensitizer (RB), followed by loading with a hydrophobic anticancer drug (a histone deacetylase inhibitor). Under NIR laser irradiation at 980 nm, UCNPs emitted UV, 540 and 650 nm luminescence bands. The UV light activated photocleavable PNBMA segments to induce the hydrophobic-to-hydrophilic transition of micelle cores, triggering a rapid drug release for NIR-controlled chemotherapy. The emitted 540 nm light could activate RB molecules to produce ^1^O_2_ for NIR-induced PDT. Further surface modification with a neuroendocrine tumor-targeting ligand allowed high tumor accumulation of micelles in a human medullary thyroid TT tumor model, thus achieving the highest antitumor efficacy.

In this section, we have introduced the utilization of UCNPs to construct NIR photoconversion responsive DDSs for cancer therapy. NIR photoactivated on-demand release of drugs can be realized via the use of UCNPs with unique intrinsic optical properties and photocleavable, photoswitchable, or photoreductive moieties. UCNPs often show excellent stability against photochemical degradation [117], and thus these photoconversion responsive DDSs have a great potential for cancer therapy. However, the relatively low quantum yields of UCNPs and in vivo safety concerns of inorganic rare elements in UCNPs need to be overcome for their further clinical applications [41].

## Conclusion and perspectives

Nanomaterial-based DDSs potentially improve the therapeutic effects and reduce side effects of chemotherapeutic drugs. However, less than 5% dosage of DDSs after systemic administration can reach tumor tissues [5], which often limits their therapeutic outcomes. The development of advanced DDSs with on-demand drug release profiles are highly desired. In this regard, NIR photoresponsive DDSs have received tremendous attention because of their unique advantages. Herein, we have summarized the recent development of NIR photoresponsive DDSs for cancer photo-chemotherapy. Based on three different photoresponsive mechanisms, these DDSs are constructed to integrate optical materials, anti-cancer drugs and responsive moieties. Upon NIR laser irradiation, optical materials convert NIR light into heat, ROS or short-wavelength light, which not only enables PTT and/or PDT, but also results in the destruction of responsive moieties for on-demand release of drugs in tumor tissues. As a result, these DDSs often afford improved therapeutic effects to reject tumors.

Although the promising achievements of NIR photoresponsive DDSs, there are several crucial issues that greatly restrict their clinical translation. First, the tissue penetration depth of NIR light has been improved compared to UV/visible light, which however is still less than 1 cm. Thus, the uses of photoresponsive DDSs are only suitable for superficial tumors such as melanoma. Since the second NIR light (NIR-II, 1000–1700 nm) shows a further increased tissue penetration depth of around 3–5 cm, development of NIR-II photoresponsive DDSs can overcome this dilemma [118]. Alternatively, the combination of light delivery technologies can be adopted to achieve a deep delivery of light sources in biological tissues [119]. Second, photon conversion efficiencies of optical components in NIR photoresponsive DDSs still need to be enhanced to improve the therapeutic efficacies upon laser irradiation at a low power density. High laser power density may induce photodamage to skill following the guidelines of American National Standard Institute (ANSI) [120]. Some strategies such as molecule acceptor doping, light-harvesting unit integration, and incorporation of different optical materials have shown great potential in amplifying the photon conversion efficiencies [37]. Third, the in vivo long-term biocompatibility and biodegradability of NIR photoresponsive DDSs is questionable and the products of photoirradiation may cause some safety concerns. To address this issue, it is necessary to systemically evaluate their biosafety in living subjects. Alternatively, efforts can be made to enhance their biodegradability and/or reduce their dimensions for a rapid clearance via renal and/or hepatic excretions [121–124]. Fourth, the variety of photoresponsive components is very limited, and their manufactures require long processing time and high cost of production, which greatly hinders the large-scale manufacturing of photoresponsive DDSs for clinical and translational applications. Exploration of facile inexpensive manufacturing methodology and/or development of new photoresponsive components is desirable to achieve their clinical translation. At last, it is often difficult to identify the tumor regions and the optimal therapeutic windows for NIR laser irradiation. Additional imaging agents can be integrated into photoresponsive DDSs to realize imaging-guided cancer photo-chemotherapy.

In addition to anticancer drugs, NIR photoresponsive DDSs can be used for on-demand release of other agents to achieve different therapeutic purposes. For example, Chang’s group reported the use of Prussian blue nanocubes to mediate photothermic activation of a tumor suppressor gene (p53) for PTT-synergistic gene therapy of tumors [125]. Via integrating photoresponsive components with immunotherapeutic molecules into a single nanoplatform, it is probable to achieve photoactivation of cancer immunotherapy using NIR photoresponsive DDSs [45]. Furthermore, the feasibility of NIR photoresponsive DDSs for the treatments of diseases other than cancer such as neurodegenerative, cardiovascular, infectious, and autoimmune diseases can be explored. Overall, with the progression of extensive research that will enable a better understanding of the current state of art, NIR photoresponsive DDSs should be available for clinical applications in the near future.

## Data Availability

Not applicable.

## Abbreviations

DDSs: Drug delivery systems
NIR: Near-infrared
ROS: Reactive oxygen species
UV: Ultraviolet
PTT: Photothermal therapy
PDT: Photodynamic therapy
PCM: Phase change material
CuS: Copper sulfide
Bi_2_S_3_: Bismuth sulfide
TPC: Tetraphenylchlorin
ICG: Indocyanine green
RB: Rose bengal
Ce6: Chlorin e6
PhA: Pheophorbide A
BODIPY: Boron dipyrromethene
CP: Conjugated polymer
SP: Semiconducting polymer
UCNPs: Upconverting nanoparticles
DOX: Doxorubicin
MCN: Mesoporous carbon nanoparticle
DSPE-PEG: 1,2-Distearoyl-sn-glycero-3-phosphoethanolamine-*N*-[amino(poly(ethylene glycol))]
MSPC: 1-Stearoyl-2-hydroxy-sn-glycero-3-phosphatidylcholine
DPPC: 1,2-Dipalmitoyl-sn-glycero-3-phosphatidylcholine
DiR: 1,1′-Dioctadecyl-3,3,3′,3′-tetramethylindotricarbocyanine iodide
ABC: Ammonium bicarbonate
DTX: Docetaxel
CPP: Cell-penetrating peptide
cRGD: Cyclic arginine-glycine-aspartic acid
PAMAM: Poly(amidoamine)
TPP: Triphenylphosphine
DSPC: 1,2-Distearoyl-sn-glycero-3-phosphocholine
LON: Lonidamine
PTX: Paclitaxel
TPZ: Tirapazamine
MSNs: Mesoporous silica nanoparticles
CA4: Combretastatin A4
EGFR: Epidermal growth factor receptor
RBC: Red blood cell
TPC: Tetraphenylchlorin
BATA: Bis-(alkylthio)alkene
BSA: Bovine serum albumin
PMAA: Poly(methacrylic acid)
PGA: Poly-ʟ-glutamic acid
β-CD: β-Cyclodextrin
HPLC: High-performance liquid chromatography
SPNs: Semiconducting polymer nanoparticles
Pt(IV): Platinum (IV)
PAA: Poly(acrylic acid)
TAT: Trans-activator of transcription
HA: Hyaluronic acid
ANSI: American National Standard Institute
